# Evaluation of stability and inactivation methods of SARS-CoV-2 in context of laboratory settings

**DOI:** 10.1007/s00430-021-00716-3

**Published:** 2021-07-01

**Authors:** Marek Widera, Sandra Westhaus, Holger F. Rabenau, Sebastian Hoehl, Denisa Bojkova, Jindrich Cinatl, Sandra Ciesek

**Affiliations:** 1 Institute for Medical Virology, University Hospital Frankfurt am Main, Goethe University, Paul-Ehrlich-Str.40, 60596 Frankfurt am Main, Germany; 2 German Center for Infection Research, DZIF, 60596 Braunschweig, Germany; 3 Branch Translational Medicine and Pharmacology, Fraunhofer Institute for Molecular Biology and Applied Ecology (IME), 60596 Frankfurt am Main, Germany

**Keywords:** SARS-CoV-2, COVID-19, Bio safety, Corona virus, Stability, Inactivation

## Abstract

The novel coronavirus SARS-CoV-2 is the causative agent of the acute respiratory disease COVID-19, which has become a global concern due to its rapid spread. Laboratory work with SARS-CoV-2 in a laboratory setting was rated to biosafety level 3 (BSL-3) biocontainment level. However, certain research applications in particular in molecular biology require incomplete denaturation of the proteins, which might cause safety issues handling contaminated samples. In this study, we evaluated lysis buffers that are commonly used in molecular biological laboratories for their ability to inactivate SARS-CoV-2. In addition, viral stability in cell culture media at 4 °C and on display glass and plastic surfaces used in laboratory environment was analyzed. Furthermore, we evaluated chemical and non-chemical inactivation methods including heat inactivation, UV-C light, addition of ethanol, acetone-methanol, and PFA, which might be used as a subsequent inactivation step in the case of insufficient inactivation. We infected susceptible Caco-2 and Vero cells with pre-treated SARS-CoV-2 and determined the tissue culture infection dose 50 (TCID_50_) using crystal violet staining and microscopy. In addition, lysates of infected cells and virus containing supernatant were subjected to RT-qPCR analysis. We have found that guanidine thiocyanate and most of the tested detergent containing lysis buffers were effective in inactivation of SARS-CoV-2, however, the M-PER lysis buffer containing a proprietary detergent failed to inactivate the virus. In conclusion, careful evaluation of the used inactivation methods is required especially for non-denaturing buffers. Additional inactivation steps might be necessary before removal of lysed viral samples from BSL-3.

**Supplementary Information:**

The online version contains supplementary material available at 10.1007/s00430-021-00716-3.

## Introduction

The coronavirus disease 2019 (COVID-19) is caused by an infection with the severe acute respiratory syndrome coronavirus 2 (SARS-CoV-2). The origin of SARS-CoV-2 outbreak was initially described in Wuhan, China [1] and rapidly established a worldwide pandemic. Globally, 126.4 million cases and 2.8 million deaths in total since the start of the pandemic have been reported by the WHO as published on 28 March 2021 [2]. Numerous experimental studies are currently being carried out, which require appropriate inactivation methods to restrict laboratory born spread among scientists and lab personnel.

SARS-CoV-2 is a spherical beta coronavirus with a size of 120 nm in diameter, which has a lipid envelope. Persistence and inactivation of coronaviruses including the highly pathogenic SARS-CoV and MERS-CoV, which emerged in the last decades, was evaluated in several publications (reviewed in [3, 4]). Accordingly, a whole series of chemical and physical inactivation methods such as UV radiation, heat inactivation and detergents are assumed to be effective inactivation methods against SARS-CoV-2 [5]. However, since human coronaviruses were shown to remain infectious on inanimate surfaces at room temperature for up to 9 days (reviewed in [3, 4]), contamination of frequently touched surfaces in laboratory settings are, therefore, a potential source of viral transmission. As shown for other enveloped viruses [6–8], it might be assumed that SARS-CoV-2 can remain infectious for much longer period of time when stored in liquid milieu at appropriate temperatures. This is the case in laboratory environments in which the condition of the sample has to be partially preserved for the subsequent molecular biological assays.

Quantitative reverse transcriptase polymerase chain reaction (RT-qPCR) is the most commonly used detection method for SARS-CoV-2, in which guanidine thiocyanate containing chaotropic buffers are efficient in disrupting viral structures. However, there are applications, in particular in molecular biology, that require incomplete denaturation of the proteins (e.g., luciferase measurements). Thus, in this study we evaluated common lysis buffers that are used in molecular biological laboratories for their ability to inactivate SARS-CoV-2. Furthermore, we investigated the stability of SARS-CoV-2 in cooled cell culture media and on touch panels on electronic devices. In addition, we evaluated the impact of temperatures, physical (UV), and chemical influences on viral infectivity as a possible method for subsequent inactivation step in the case of insufficient inactivation by the lysis buffer.

## Materials and methods

### Cell culture and virus propagation

Caco-2 and Vero cells were cultured in minimum essential medium (MEM) supplemented with 10% fetal calf serum (FCS), 100 IU/ml of penicillin and 100 µg/ml of streptomycin. Viral titers of SARS-CoV isolate HK or FFM1 [9] and SARS-CoV-2 isolate (Frankfurt 1, FFM1) [10, 11] were determined by tissue culture infection dose 50 (TCID_50_). For virus propagation, Caco-2 or Vero cells were seeded in a 96-well plate and inoculated with SARS-CoV or SARS-CoV-2 using a MOI of 0.1 [11]. After 60 min at 37 °C and 5% CO_2_ cells were rinsed with PBS, supplemented with fresh medium, and incubated until harvest. Influenza isolates PR8 (H1N1) [ATCC-VR-1469; p1; Titer: 1.1 × 10^7^] and Victoria (H3N2) [ATCC-VR-822; p5; Titer: 1.4 × 10^7^] were prepared by infecting MDCK cells. Cell free virus aliquots were stored at −80 °C. All infectious work was performed under biosafety level 3 (BSL-3) conditions according to the Committee on Biological Agents (ABAS) and Central Committee for Biological Safety (ZKBS).

### *Determination of SARS-CoV-2 infectivity by TCID*_*50*_

Infectious titer of SARS-CoV-2 supernatant were determined by an end-point limiting dilution assay as 50% tissue culture infectious dose (TCID_50_/ml) in confluent cells in 96-well microtiter plates. Briefly, Caco-2 cells were seeded onto a 96-well plate and infected with untreated or treated SARS-CoV-2 containing supernatant in an initial 1:10 dilution (quadruplicates). Titration was performed on 96-well plate with ten serial 1:10 dilutions. Viral titer was determined 48 h post infection as 50% TCID_50_ as described by Spearman and Kaerber [12, 13]. To analyze inhibition capacity of different inactivation methods viral titers were normalized to the untreated control and reduction factor was calculated as described elsewhere [14]. All infection experiments were performed with initial viral titer of 1 × 10^6^ TCID_50_/ml if not indicated differently.

### Chemical inactivation of cell culture supernatants

To test different chemical compounds to inactivate SARS-CoV-2, virus containing supernatant was mixed 1:1 or 1:10 with lysis buffers or fixation solutions, respectively. As adapted from previously published procedures [15], the mixture was incubated for 10 min at room temperature before further processing. Testing different lysis buffers, compound-virus mixture was further diluted 1:1000 to avoid cytotoxic effect before added to the seeded Caco-2 cells. Viral load and inactivation capacity were determined by RT-qPCR and CV staining, respectively. Analyzing different fixation solutions, compound-virus mixture was further diluted 1:100 before added to the seeded Vero cells. Viral titers and inactivation capacity were determined by RT-qPCR and compared to viral control. Before use, Western blot (WB) lysis buffer (20 mM TRIS/HCl pH 7.5, 150 mM NaCl, 10 mM NaPPi, 20 mM NaF, 1% Triton-X, 1.9 M glycine, and 250 mM TRIS base) was supplemented with cOmplete™ protease inhibitor cocktail tablets (Roche).

### Physical inactivation of cell culture supernatants

Analyzing physical parameters to inactivate SARS-CoV-2, virus containing supernatant was treated either at different temperatures or with UV-C light or was applied to different surfaces and stored for a distinct period of time. For surface inactivation SARS-CoV-2 was applied to cell culture dishes (polystyrole; plastic), used smartphone display glass, and used protection film and dried at ambient temperature. Of note, the used surfaces were not cleaned prior to the experiments to maintain an environment close to real-world conditions. Dried virus spots were incubated for 6 h or 5 days at ambient temperature before wiped off with a PBS soaked swab (2 cm^2^) and eluted in culture medium. Testing different temperatures for inactivation of SARS-CoV-2 virus containing supernatant was incubated on a thermo shaker for defined time at 56, 60 or 90 °C. In addition, determination of viral load was performed by infecting Vero cells and measuring gene copies/reaction by RT-qPCR. Finally, SARS-CoV-2, SARS-CoV, and Influenza A containing supernatant was applied to cell culture dishes and dried. Dried virus spots were treated with UV light of different light sources. “UVA-Cube” and UV-C LED (Hönle AG, Germany) both contain a UV-C light source (LED Spot 100 IC / HP IC), which was set to *E* = 8.8 mW/cm^2^ (365–460 nm) with a fixed distance of 2 cm. Benchtop UV light was emitted using a laminar flow (Holton LaminAir, Heto-Holten, Denmark). After irradiating for a defined time spots were eluted in culture medium. Viral load and inactivation capacity were determined by TCID_50_ and crystal violet (CV) staining, respectively.

### Rotitest vital viability assay

To analyze the viability of cells that were exposed to chemically or physically inactivated SARS-CoV-2, the Rotitest Vital Kit (Carl Roth GmbH + Co. KG, Karlsruhe, Germany) was used. Briefly, cells were seeded in 96-well plates and incubated with a 1:1000 dilution of each inactivating compound in culture medium. The assay was performed in triplicates per sample. After 48 h of incubation at 37 °C and 5% CO_2_, Rotitest Vital solution was added as described by the manufacturer. The intracellular dehydrogenase activity was analyzed using a multimode reader at 450 nm.

### RT-qPCR analysis and detection of SARS-CoV-2 genomic RNA

For detection of intracellular and extracellular SARS-CoV-2 genomic RNA, primers M-475-F (5'-TGTGACATCAAGGACCTGCC-3') and M-574-R (5'-CTGAGTCACCTGCTACACGC-3') were used together with the Fam-BHQ1 dual-labeled probe M-507-P (5'-TGTTGCTACATCACGAACGC-3') as described previously [11]. For normalization to intracellular mRNA level, human GAPDH was measured using primers GAPDH-fwd (5'-TGCACCACCAACTGCTTA) and GAPDH-rev (5'-GGATGCAGGGATGATGTTC-3').

### Determination of SARS-CoV-2 inactivation by crystal violet staining

Inactivation of SARS-CoV-2 by chemical and physical methods was (when indicated) determined by crystal violet staining (CV). Therefore, Caco-2 cells were seeded on a 96-well plate infected with untreated or treated SARS-CoV-2 supernatant and cultured for 48 h at 37 °C, 5% CO_2_. Cells were fixed using 3% paraformaldehyde (PFA) and incubated for 20 min at room temperature. Staining was performed with 0.1% CV for another 20 min at room temperature. Staining solution was removed and plates were dried overnight. CV was dissolved with 100% ice-cold methanol and analyzed using a multimode reader at 560 nm. To determine inactivation capacity samples were normalized to untreated control.

### Statistical analysis

If not indicated differently all infection experiments were repeated in three independent experiments. Data analysis was performed in Microsoft Excel and GraphPad Prism 6 (GraphPad Software, USA). Statistical significance compared to untreated control was determined using unpaired Student’s *t *test on non-log-transformed data. Asterisks indicated *p *values as *p < 0.05, **p ≤ 0.01, and ***p ≤ 0.005.

## Results

### SARS-CoV-2 is stable for several weeks

**Fig. 1:**
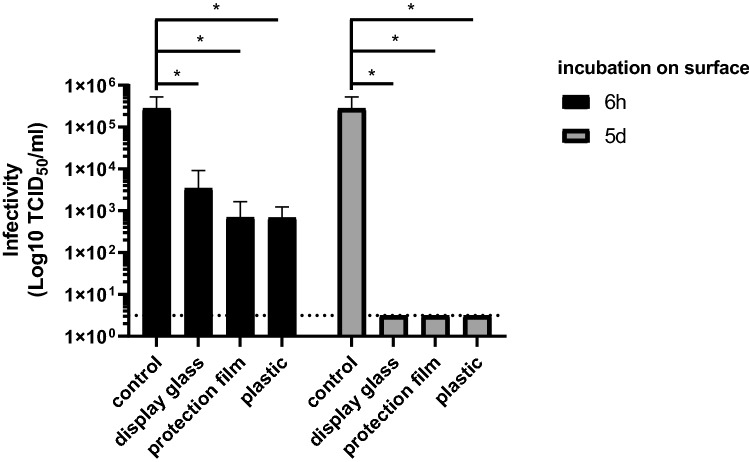
Stability of SARS-CoV-2 on surfaces of electronic devices. Stability of SARS-CoV-2 on surfaces with and without protection film. TCID_50_ using Caco-2 cells was performed to determine viral infectivity (microscopical CPE readout). Representative experiment performed in quadruplicates. Error bars indicate SD from four technical replicates

Coronaviruses were shown to remain infectious on surfaces commonly used in laboratories for a considerably period of time [16]. We were particularly interested in the persistence of SARS-CoV-2 on frequently touched surfaces in laboratories and examined how long SARS-CoV-2 might be stable on touch panels of electronic devices. For this purpose, we coated plastic as well as a touchscreen glass display of a mobile phone with and without protective film with SARS-CoV-2 strain FFM1 [10, 11] using moderate concentration of 2.8 × 10^5^ TCID_50_/ml. After a short and long incubation time at room temperature corresponding to one working day (6 h) and one working week (5 days), the surfaces were wiped off with a PBS soaked swab and the viruses were eluted in culture medium and tested quantitatively for infectivity. After 6 h, we observed a decrease in TCID_50_ of 1.91 log on the display and of approx. 2.6 log for displays with a protective glass and for plastic surfaces (Fig. 1; Table 1). After 5 days, we were unable to recover infectious virus particles on any of the surfaces indicating a minimal reduction factor of > 4.95 log. These data were in agreement with prior studies showing that SARS-CoV-2 is relatively stable on smooth surfaces like glass and plastic [17, 18]. Of note, a biphasic decay of infectious SARS-CoV-2 was observed with a long-term half-life (*t*½) of 4.8 h on glass and between 6.8 and 11.4 h on plastic [17, 18].

**Table 1:** Stability of SARS-CoV-2 on surfaces in laboratory environment

Incubation time	Surface	Virus titer (TCID_50_/ml [log10] ± SD)	Minimal reduction factor (log10)	*p *value
Viral control		5.45 ± 5.39	0.00	–
6 h	Display glass	3.54 ± 3.75	1.91	0.0377
	Protection film	2.84 ± 2.98	2.60	0.0357
	Plastic	2.84 ± 2.74	2.61	0.0357
5 days	Display glass	0.5 ± 0.0	4.95	0.0352
	Protection film	0.5 ± 0.0	4.95	0.0352
	Plastic	0.5 ± 0.0	4.95	0.0352

Common laboratory surfaces plastic and touchscreen displays with and without protection glass were contaminated with infectious SARS-CoV-2. Recovered virus was tested for infectivity performing TCID_50_ assays. Detection limit was 0.5 TCID_50_/ml (log10)

To investigate how long SARS-CoV-2 viruses are stable in cell culture media at 4 °C, we thawed different stock aliquots of a SARS-CoV-2 (which were cryo-conserved at −80 °C, 1 × 10^8^ TCID_50/ml_) at weekly intervals and stored them at 4 °C in cell culture medium for up to 160 days. Remarkably, using TCID_50_ even after 160 days we were still able to detect considerable quantities of infectious virus (> 1 × 10^5^ TCID_50_/ml, < 3 log reduction, data not shown), which was in line with previous findings demonstrating long-term survival under 4 °C [18, 19].

These data indicate that it is particularly important to inactivate contaminated samples and instruments before continue working under less stringent safety requirements.

### Effectiveness of lysis buffers at inactivating SARS-CoV-2

**Fig. 2:**
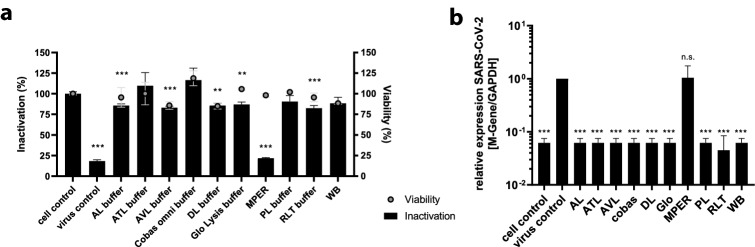
Inactivation of SARS-CoV-2 by lysis buffers commonly used in molecular biology *laboratories.* **a** Crystal violet staining of Caco-2 cells previously infected with virus pre-incubated with the indicated lysis buffer. Cell viability was determined by commercial Rotitest Vital Assay measuring OD_450_. Cells were incubated with a mixture of cell culture medium and the depicted lysis buffer in the given concentration used for virus inactivation. For the untreated control no viability assay was performed to avoid handling with infectious samples outside the safety cabinet. Samples were diluted 1:1000 to avoid cell toxicity. **b** RT-qPCR analysis of intracellular RNA obtained from infected cells Caco-2 cells showing the relative expression of SARS-CoV-2 RNA targeting M-Gene. Values were normalized to cellular GAPDH expression. Error bars indicate SD from the mean of representative experiment performed in triplicates. Virus control indicates SARS-CoV-2 infected cells without pre-incubation. Cell control indicates uninfected Caco-2. AL (Qiagen), ATL (Qiagen), AVL (Qiagen), cobas omni buffer (Roche) and RLT (Qiagen) contain guanidine thiocyanate. DL (in-house) contains IGEPAL CA-630. M-PER (Thermo Scientific) lysis buffer containing a proprietary detergent. WB (in-house) and Glo buffer (Promega Glo-Lysis buffer) contains Triton-X. PL (lysis juice, p.j.k) is a detergent free proprietary lysis buffer

Molecular biological detection methods require specific lysis methods to ensure subsequent partly enzymatic reactions. Buffers containing guanidine thiocyanate have proven useful for the isolation of viral nucleic acids and PCR-based analysis. We have, therefore, tested a set of six common lysis buffers for nucleic acid extraction whereof three are also used in routine diagnostic. Buffers AL, ATL, and AVL (Qiagen, Hilden, Germany) and the Cobas Omni buffer (Roche, Germany) are commonly used to isolate nucleic acids from cell culture supernatants and patient material such as serum, blood, plasma, but also throat swab samples. The buffer RLT (Qiagen), however, is mainly used for research purpose for the isolation of total cellular RNA from infected cells. SARS-CoV-2 containing cell culture supernatants were mixed with lysis buffers (1:1) and diluted after 10 min of incubation. To evaluate the remaining infectivity viral outgrowth assays were performed in Caco-2 cells and after 48 h total RNA from infected cells was isolated for further analysis. To control that the readout was not affected by cell toxicity issues, in parallel we performed a cell viability assay (Fig. 2a). To monitor virus production, we additionally carried out an RT-qPCR targeting the SARS-CoV-2 M gene. As shown in Fig. 2 all tested buffers containing guanidine isothiocyanate including the cobas omni buffer (Roche) were able to completely inactivate samples containing SARS-CoV-2 (Table 2).

**Table 2:** Inactivation of SARS-CoV-2 by lysis buffers commonly used in laboratory settings

Lysis buffer	Inactivation (%) (crystal violet staining)	Relative expression M-Gene (RT-qPCR)	*p *value (RT-qPCR)
cell control	n. d.	0.062	≤ 0.001
virus control	18	1.000	–
AL buffer	86	0.062	≤ 0.001
ATL buffer	109	0.062	≤ 0.001
AVL buffer	83	0.062	≤ 0.001
Cobas omni buffer	116	0.062	≤ 0.001
DL buffer	86	0.062	≤ 0.001
Glo lysis buffer	87	0.062	≤ 0.001
M-PER	**22**	**1.043**	**0.922**
PL buffer	91	0.062	≤ 0.001
RLT buffer	82	0,045	≤ 0.001
Western blot lysis buffer	88	0.062	≤ 0.001

Percent inactivation relative to OD_570_ in Crystal Violet staining, normalized to cell control (without virus). RT-qPCR data showing relative SARS-CoV-2 M gene expression for treated samples related to the virus control

The IGEPAL CA-630 (MP Biomedicals Germany GmbH, Eschwege, Germany) containing direct lysis (DL) buffer was developed to avoid expensive and time-consuming RNA extraction procedure while concomitantly inactivating infectious samples prior to PCR analysis. DL buffer was found to efficiently inactivate SARS-CoV-2 (Table 2; Supplementary Fig. 1). Next, we also tested lysis buffers that are commonly used to analyze protein samples. We used Triton-X containing buffers for Western blot analysis and luciferase-based assay. The latter have the critical property of maintaining protein function to allow enzymatic reactions with light emission. The buffers used, therefore, have a membrane-permeabilizing, but incompletely denaturing effect on cells. For Western blot lysis buffer and the commercially available Glo-Lysis buffer (Promega, Walldorf, Germany) as well as the PL buffer (lysis juice; p.j.k., Kleinblittersdorf, Germany) we were able to demonstrate complete inactivation (Table 2, Supplementary Fig. 1). Importantly, Mammalian Protein Extraction Reagent (M-PER, ThermoFisher) was not able to inactivate SARS-CoV-2 (Fig. 2; Table 2). As determined using crystal violet staining, only 22% of the cells remained after infection, which was comparable to the untreated virus control (18%). These data were confirmed by RT-qPCR since the relative SARS-CoV-2 M gene expression in cells infected with M-PER treated virus was not statistically different from the virus control (1.043-fold change to the virus control).

These findings highlight the fact that careful evaluation of the used inactivation methods are required and that additional inactivation steps might be necessary to treat samples for safe processing.

### Physical inactivation effectively eliminates infectious SARS-CoV-2

Since not all lysis buffers were able to completely inactivate SARS-CoV-2, we have further tested physical and chemical inactivation methods that can be applied subsequently. First, we evaluated the effects of heat inactivation on SARS-CoV-2 infectivity and heated cell culture supernatants at different temperatures with different incubation times. To monitor the temperature of the liquid that has to be inactivated, we generated heat curves (Supplementary Fig. 2, Supplementary Tables 1–2). Subsequently, the treated supernatants were used to infect Caco-2 cells. After 48 h cell culture supernatants were harvested and subjected to RT-qPCR to evaluate viral outgrowth (Table 3). We found that heat inactivation by placing the tube (500 µl in a 1.5 ml vessel) from ambient temperature (approx. 23 °C) to a pre-warmed heating block for 5 min at 56 °C was not sufficient (0.15 log), while increasing the incubation time to 30 min was a highly effective method of inactivation (4.35 log). Already 5 min at 60 °C drastically reduced the infectivity of the virus suspension by 4.35 log. Short incubation time of 1 min at 90 °C also achieved a very effective 4.28 log reduction in SARS-CoV-2 viral load. Of note, the deviation in the log reduction between short and long incubation times may have occurred due to experimental variations. A significant decrease of  > 3.7 log steps was considered as sufficient for viral inactivation.

**Table 3:** Heat inactivation, log reduction

Temperature (°C)	Incubation time (min)	Virus load (copies/reaction)	Minimal reduction factor (log10)	*p *value
Viral Control		6.15 × 10^7^	0.00	–
56	5	4.31 × 10^7^	0.15	0.185
	30	2.74 × 10^3^	4.35	0.003
60	5	3.48 × 10^3^	4.25	0.003
	30	6.23 × 10^3^	3.99	0.003
90	1	3.22 × 10^3^	4.28	0.003
	5	1.19 × 10^4^	3.71	0.003

Virus load was determined via RT-qPCR targeting SARS-CoV-2 M gene with a detection limit of 2 × 10^3^ copies/reaction. A reduction > 3.7 log_10_ was considered as efficient

Next, we evaluated which chemical disinfectant and fixation solutions are appropriate to inactivate SARS-CoV-2. We diluted virus containing supernatants with cell culture media (1:10), mixed the dilution to the testing compound, and incubated for 10 min at ambient temperature. Acetone/methanol (40:60), ethanol (70%), and paraformaldehyde (PFA, 3%) were found suitable to completely inactivate the virus (Table 4). However, ethanol/PBS (50%, 1:1) did not inactivate SARS-CoV-2, which was in line with previously published data [5].

**Table 4:** Fixation of SARS-CoV-2

Fixation solution	Virus load (copies/reaction [Log 10] ± SD)	Minimal reduction factor (Log10)	*p *value
Viral control	7.63 ± 7.23	0.00	–
Acetone/methanol (40:60)	3.34 ± 2.96	4.38	0.012
70% ethanol	3.65 ± 3.50	4.02	0.012
50% ethanol/PBS (1:1)	7.69 ± 6.85	0.00	0.568
3% paraformaldehyde	3.82 ± 3.05	3.81	0.012

Virus load was determined via RT-qPCR targeting SARS-CoV-2 M gene with a detection limit of 3.3 copies/reaction (Log10). A reduction > 3.7 log_10_ was considered as efficient

**Fig. 3:**
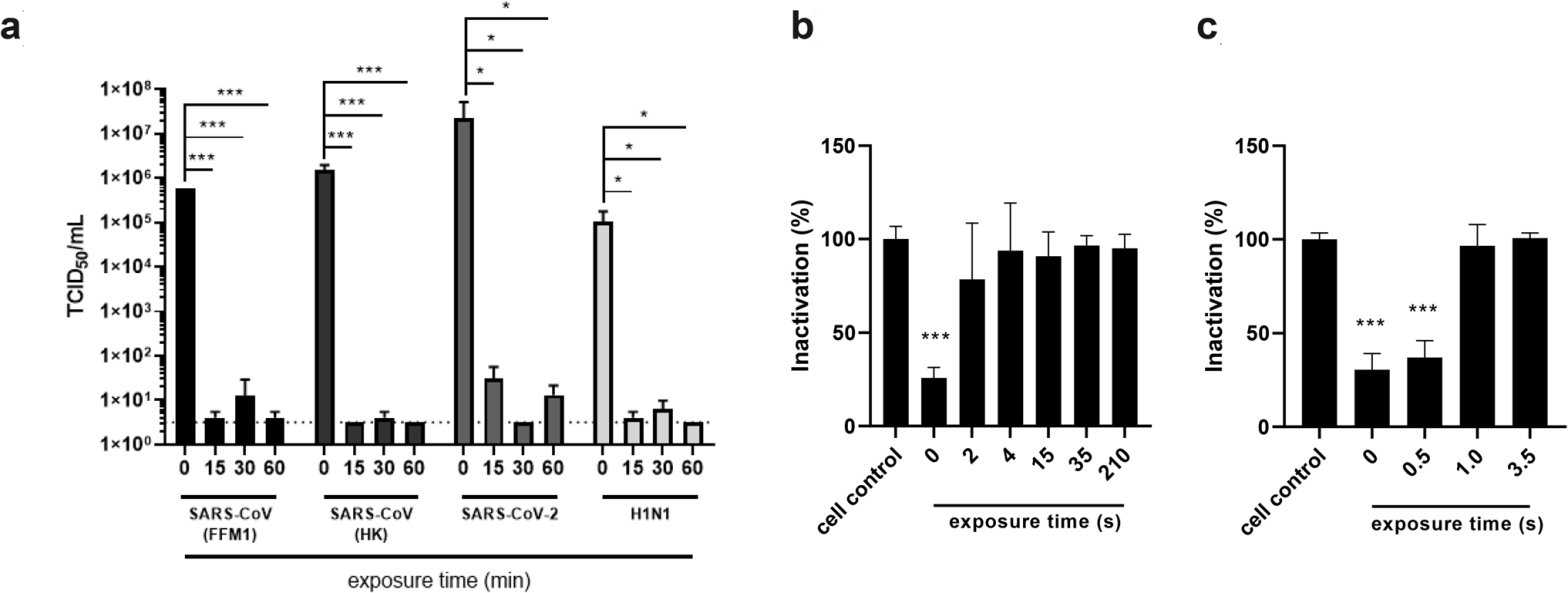
Inactivation of SARS-CoV-2 using UV-C light. **a** Quantification of SARS-CoV-2 using microscopical CPE based TCID_50_ in Caco-2 cells. After exposure to UV-C light in the safety cabinet for the indicated exposure time SARS-CoV-2 was used to infect Caco-2 cells. SARS-CoV-strains FFM1 [9] and Hong Kong (HK), SARS-CoV-2 strain FFM1 [10, 11], H1N1 (PR8). Cristal violet staining of Caco-2 cells infected with UV-irradiated SARS-CoV-2. UV-C discharge lamp (**b**) and UV-C LED (**c**) were used as light source for the indicated exposure time. s, seconds. Error bars indicate SD from the mean of three independent experiments

Finally, we investigated the influence of UV light on the stability of SARS-CoV-2. For comparison, we included two different strains of SARS-CoV (from 2003) and also H1N1 Influenza A virus (IAV) as a representative of a seasonally recurring respiratory pathogen. To mimic daily laboratory decontamination routines, we exposed SARS-CoV-2 contaminated samples in a biological safety cabinet to UV light and irradiated for 15–60 min. We found that 15 min were already highly efficient and 30 min as well as 60 min completely inactivated SARS-CoV-2 infectivity. In addition, we used two different UV light sources with a discharge lamp (low pressure radiation chamber) or a highly potent LED UV light to inactivate contaminated surfaces. We compared different exposure times ranging from 2 to 210 s for UV-C discharge lamp and 0.5–3.5 s for UV-C LED with fixed distances of 20–40 mm to the light source, respectively. Using UV light from the discharge lamp, 2 s exposure time resulted in a minimal reduction factor of 3.5 log while after 4 s no infectious SARS-CoV-2 was detected in the viral outgrowth assay (Fig. 3; Table 5). One second UV-C LED was sufficient to reduce viral infectivity by 3.9 log. 3.5 s completely reduced viral titers > 4 log.

**Table 5:** Inactivation of SARS-CoV-2 by UV irradiation

Light source	Exposure time (s)	Virus titer (TCID_50_/ml [log10] ± SD)	Minimal reduction factor (Log10)	*p *value
UV-C discharge lamp	Viral control	4.51 ±	–	–
	2	1.0 ±	3.5	≤ 0.001
	4	0.5 ±	4.0	≤ 0.001
	15	0.5 ±	4.0	0.035
	35	0.5 ±	4.0	0.035
	210	0.5 ±	4.0	0.035
UV-C LED	Viral control	4.73 ±	–	–
	0.5 s	3.01 ±	1.7	0.179
	1.0 s	0.8 ±	3.9	0.174
	3.5 s	0.5 ±	4.0	0.175

Quantification of SARS-CoV-2 using microscopical CPE based TCID_50_. After exposure to UV-C light for the indicated exposure time SARS-CoV-2 was inoculated on Caco-2 cells. Detection limit: 3.2 × 10^0^ TCID_50/ml_. A reduction > 3.7 log_10_ was considered as efficient

These data demonstrate that UV light exposure is a suitable method for complete inactivation of SARS-CoV-2 contaminated material. In particular, irradiation with UV-C LED light might also represent a fast and reliable inactivation method for contaminated material and surfaces also in laboratory settings.

## Discussion

Enveloped viruses are less resistant to chemical and physical environmental influences compared to non-enveloped viruses [5, 20]. Depending on air temperature and relative humidity [21] coronaviruses are stable for up to 9 days on inanimate surfaces like metal, glass or plastic [4, 22]. In this study mid-term and long-term storages revealed a high stability of the virus during liquid storage over several weeks. We have found that even after 160 days of storage at 4 °C in cell culture medium, we still found considerable quantities of infectious virus, which corresponds to an estimated reduction < 3 log TCID_50/ml_. Data from an previously published study by Chin et al. support this observation as a reduction of infectious titer of 0.7 log was found after 14 days storage at 4 °C. Of note, the authors also described a biphasic reduction of SARS-CoV-2 on surfaces, since the estimated t½ was significantly shorter in the first than in the following hours [18]. In particular, on glass the authors stated a short t½ (0–3 h) of 1.2 h and a long t½ (3 h—2 days) of 4.8 h. On plastic, the short t½ (0–6 h) was comparable (1.6 h) to glass but the long-term t½ (6 h–4 days) was considerable higher (11.4 h). Our data have shown that SARS-CoV-2 might be slightly more stable on touchscreen display glass (without protective film), which is a technically modified glass surface. However, the difference to plastic was not significant. This observation is supported by a recent study by Liu et al. [23] demonstrating a comparable stability on glass and plastic, while no detection was possible after four days of incubation. These data suggest that laboratories must be certain that their inactivation methods for samples and disinfection of equipment used for subsequent research purposes outside a BSL-3 laboratory are sufficient. For the latter, SARS-CoV-2 can be efficiently inactivated by surface disinfection procedures [4].

For the detection of nucleic acids or proteins, strong lysis buffers are used for complete denaturation, however, several research methods in molecular biology require functional proteins for enzymatically catalyzed reactions. In this manuscript, we tested several lysis buffers and other chemical and physical inactivation methods that would allow downstream analysis of SARS-CoV-2 infection outside of the BSL-3 laboratory. In comparison to the Triton-X-containing lysis buffers used for enzyme based assays (e.g., luciferase-based measurements), we found that the commercially available M-PER buffer (Thermo Scientific) was not sufficient to inactivate SARS-CoV-2. M-PER is a proprietary detergent in 25 mM bicine buffer (pH 7.6) used for enzyme based reporter protein assays and immunoassays as well as protein purification. While bicine is an organic compound used as a buffering agent the exact composition of M-PER is not publicly known. However, according to the manufacturer, M-PER reagent has been tested on different cell lines showing complete lysis of adherent cells. Even if the manufacturers describe an efficient and fast cell lysis, in our experiments SARS-CoV-2 was able to withstand this mild detergent.

In agreement with previously published studies we found that most physical and chemical inactivation methods were effective and might be used as a second inactivation step. In particular, our results were in line with studies showing high temperature [24], alcohols [5], UV irradiation [25], and lysis by detergents were effective in inactivate corona virus [14, 15]. However, in agreement with previously published data on SARS-CoV [15], fixation with PBS/ethanol (EtOH 50%; 1:1) over 5 min was not sufficient to eliminate infectious virus.

We found that heat inactivation for 30 min at 56 °C, 5 min at 60 °C, and 1 min at 90 °C were highly effective and reduced the infectivity of the virus suspension by more than 4 log. Thus, heat inactivation is suitable for direct SARS-CoV-2 inactivation but also for subsequent treatment of samples that are in a suboptimal lysis buffer like M-PER. Importantly, it is essential that the heating block is preheated and that the time is adapted to the volume to be inactivated (Supplementary Fig. 2, Supplementary Tables 1 and 2).

UV irradiation of infected cell culture samples was very effective in our study, but varied between the methods. Our results were partly consistent with previously published results recently reviewed by Derraik and colleagues [26]. In a study by Darnell et al., an experimental setup similar to our setup was performed, and revealed that already 1 min UV-C (distance from source 3 cm) irradiation led to a decrease in infectious SARS-CoV titers and that 6 min irradiation led to an almost complete inactivation [27]. After 15 min of UV-C irradiation no detection of infectious viruses was possible. These data are consistent with our observation that after 15 min UV-C irradiation in a sterile workbench more than 4 log loss in infectivity was observed for SARS-CoV-2. After irradiation with a highly potent UV-C source after 35 s and with a UV-C LED even after 1 s the vast majority of viruses were already inactivated. The usual radiation occurs within the safety cabinets after work. Here, 15 min were already highly effective to eliminate infectious virus. In most laboratories, an exposure time of 30 min is suggested after infectious work. Thus, after correct use, inactivation of SARS-CoV-2 can be assumed, however, the physical lifespan of the light source must not be exceeded to generate sufficient emission. For fast inactivation of small surfaces, we evaluated two different UV-C light sources and found that UV-C discharge lamp with a radiation duration of 4 s was sufficient to inactivate SARS-CoV-2. The use of the high-potency UV-C LED light already results after 1 s. Although, all tested UV light source were UV-C rays the distance between light source and sample plays a critical role to define irradiation time. Furthermore, it has to take in account that lifespan of conventional UV lamps used in safety cabinets ranges between 6000 and 8000 h. Therefore, intensity and effectiveness of the UV light is decreasing over time and when reaching maximum of expected useful life. In conclusion, irradiation with UV-C LEDs thus represents a highly effective inactivation method for contaminated surfaces.

This study has limitations that need to be considered. To maintain real-lab-settings, we performed the stability evaluation with daily used virus stocks thawed for routine infectious experiments. Since the stocks in use had different lifetimes, longer standing times at ambient temperature could possibly have influenced viral stability. Hence we quantitatively cannot compare the samples with each other and also statistical evaluation was not applicable. Far more replicates under more controlled conditions would be necessary here. Nonetheless, even after 160 days we still found high-titer virus that has to be adequately inactivated by appropriate lysis buffer. These data are, therefore, important as a derivation for the inactivation tests. A further limitation of this study is based on the fact that inactivation efficiency largely depends on the initial virus load and the reaction volume. Especially in the case of heat inactivation, the heating time of the respective medium must be considered (Supplementary Fig. 1, Supplementary Tables 1, 2). In addition, the higher the virus titer, the longer the inactivation time is necessary for complete inactivation.

SARS-CoV-2 is able to replicate in susceptible cells lines like Caco-2 and Vero cells both yielding high viral titers in the cell culture supernatants. The main difference is the ability to form a CPE, which is definitely more pronounced in Caco-2 cells [10]. However, in this study we did not match results obtained from different experiments with dissimilar cell lines but rather compare heat inactivation conditions in a defined setup using a specific cell line. Since the respective cell line was used for readout purpose, only the reduction factor which mirrors the heat inactivation efficiency is relevant.

In conclusion, most lysis buffers commonly used in research laboratories were suitable to inactivate SARS-CoV-2. However, since the non-denaturating M-PER buffer failed to inactivate SARS-CoV-2, subsequent inactivation methods as heat inactivation or UV light must be performed afterwards. Alternatively, alcohols (except EtOH 1:1), acetone–methanol, or PFA have to be added. All buffers and inactivation methods must be carefully evaluated for their property to inactivate SARS-CoV-2 to protect laboratory personnel.

## Supplementary Information

Below is the link to the electronic supplementary material.

Supplementary file1 (PDF 2315 KB) Supplementary Figure 1 SARS-CoV-2 inactivation with Triton-containing lysis buffer (PL) and IGEPAL-630 containing buffer (DL). Crystal violet staining of Caco-2 cells infected with SARS-CoV-2 previously inactivated with the indicated buffer. Virus and lysis buffer (1:1) were incubated for 15 min at ambient temperature. Samples were diluted as indicated in brackets to avoid cell toxicity

Supplementary file2 (PDF 272 KB) Supplementary Figure 2 Heating curve of liquids in common laboratory reaction vessels. To minimize evaporation, 500 µl (black circles and line) or 2000 µl (grey squares and line) glycerol in a 1.5 ml or 2 ml reaction vessel, respectively, were placed in a preheated heating block. The increase in temperature was monitored by a thermometer immersed in glycerol. Mean values (n=3) were rounded up to the nearest integer. Error bars indicate standard deviation

Supplementary file3 (DOC 17 KB)
