# Supplementary figures and images for: Evaluation of stability and inactivation methods of SARS-CoV-2 in context of laboratory settings
Source: Med Microbiol Immunol. 2021 Jul 1;210(4):235–44. doi: 10.1007/s00430-021-00716-3 (PMC8245923; doi:10.1007/s00430-021-00716-3)

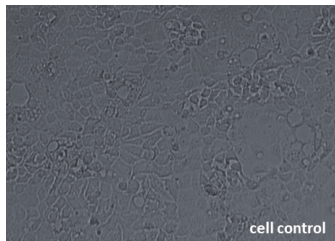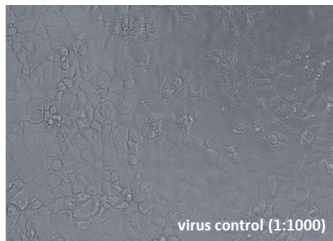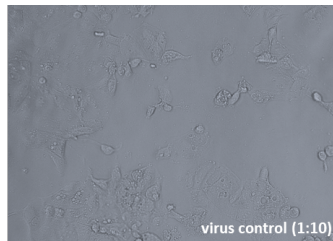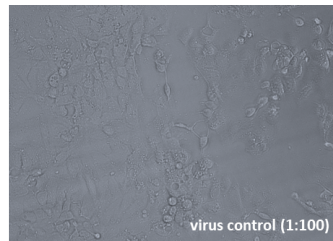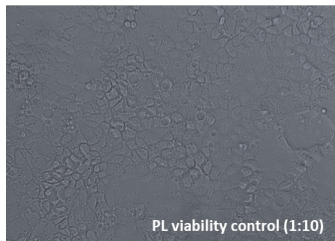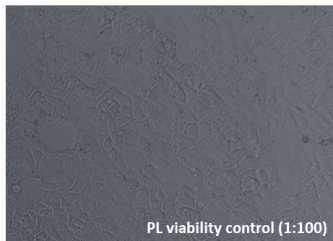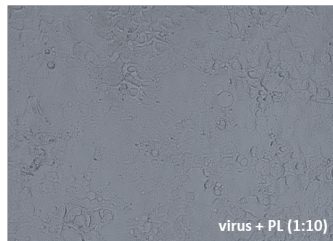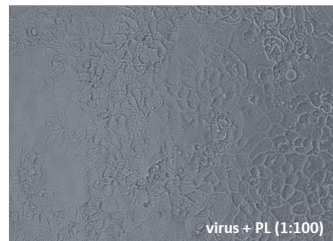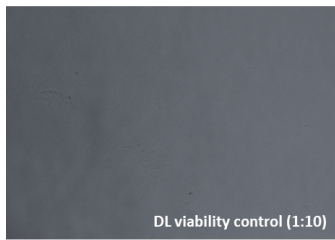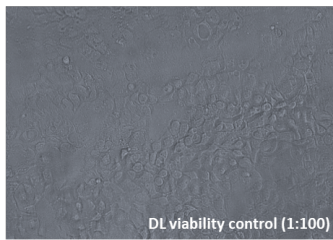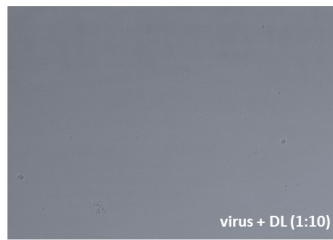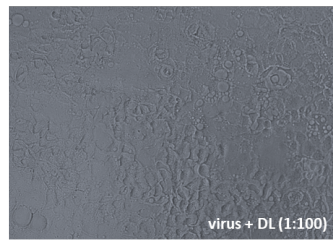

Supplement: Supplementary file 1 — Supplementary file1 (PDF 2315 KB) Supplementary Figure 1 SARS-CoV-2 inactivation with Triton-containing lysis buffer (PL) and IGEPAL-630 containing buffer (DL). Crystal violet staining of Caco-2 cells infected with SARS-CoV-2 previously inactivated with the indicated buffer. Virus and lysis buffer (1:1) were incubated for 15 min at ambient temperature. Samples were diluted as indicated in brackets to avoid cell toxicity [file 430_2021_716_MOESM1_ESM.pdf]

56°C

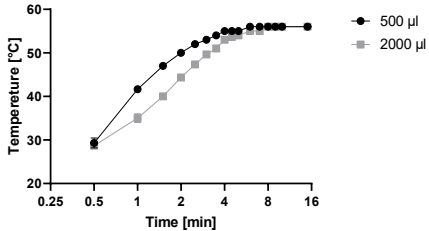

60°C

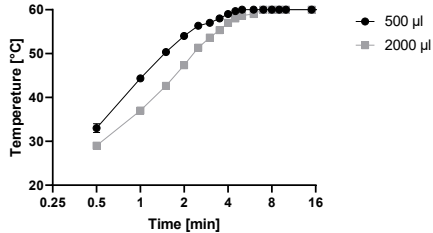

90°C

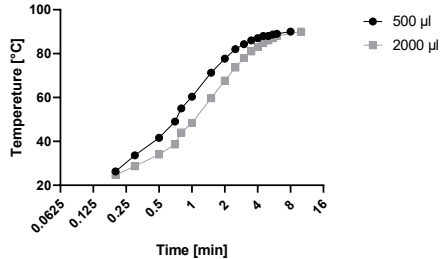

Supplement: Supplementary file 2 — Supplementary file2 (PDF 272 KB) Supplementary Figure 2 Heating curve of liquids in common laboratory reaction vessels. To minimize evaporation, 500 µl (black circles and line) or 2000 µl (grey squares and line) glycerol in a 1.5 ml or 2 ml reaction vessel, respectively, were placed in a preheated heating block. The increase in temperature was monitored by a thermometer immersed in glycerol. Mean values (n=3) were rounded up to the nearest integer. Error bars indicate standard deviation [file 430_2021_716_MOESM2_ESM.pdf]
