# Supplementary material for: Evaluation of stability and inactivation methods of SARS-CoV-2 in context of laboratory settings
Source: Med Microbiol Immunol. 2021 Jul 1;210(4):235–44. doi: 10.1007/s00430-021-00716-3 (PMC8245923; doi:10.1007/s00430-021-00716-3)
Supplement: Supplementary file 3 — Supplementary file3 (DOC 17 KB) [file 430_2021_716_MOESM3_ESM.docx]

## **Supplementary Tables:**

**Supplementary Table 1:** Heating curve of liquids in common laboratory 1.5 and 2 ml reaction vessels placed in a heat block pre-warmed to 56°C and 60°C, respectively. The increase in temperature was monitored by a thermometer immersed in 500 µl (1.5 ml vessel) and 2000 µl (2 ml vessel) glycerol, respectively. Experiments were performed in triplicates. Mean values were rounded up to the nearest integer.

| **Time (min)** | **Temperature (°C)**  500 µl at 56°C | **Temperature (°C)**  2000 µl at 56°C |  |  |  | **Temperature (°C)**  500 µl at 60°C | **Temperature (°C)**  2000 µl at 60°C |
| --- | --- | --- | --- | --- | --- | --- | --- |
| 0 | 23 | 23 |  |  |  | 23 | 23 |
| 0.5 | 29 | 29 |  |  |  | 33 | 29 |
| 1 | 42 | 35 |  |  |  | 44 | 37 |
| 1.5 | 47 | 40 |  |  |  | 50 | 43 |
| 2 | 50 | 44 |  |  |  | 54 | 47 |
| 2.5 | 52 | 47 |  |  |  | 56 | 51 |
| 3 | 53 | 50 |  |  |  | 57 | 54 |
| 3.5 | 54 | 51 |  |  |  | 58 | 55 |
| 4 | 55 | 53 |  |  |  | 59 | 57 |
| 4.5 | 55 | 54 |  |  |  | 60 | 58 |
| 5 | 55 | 54 |  |  |  | 60 | 59 |
| 6 | 56 | 55 |  |  |  | 60 | 59 |
| 7 | 56 | 55 |  |  |  | 60 | 60 |
| 8 | 56 | 56 |  |  |  | 60 | 60 |
| 9 | 56 | 56 |  |  |  | 60 | 60 |
| 10 | 56 | 56 |  |  |  | 60 | 60 |
| 15 | 56 | 56 |  |  |  | 60 | 60 |
| 20 | 56 | 56 |  |  |  | 60 | 60 |
| 25 | 56 | 56 |  |  |  | 60 | 60 |
| 30 | 56 | 56 |  |  |  | 60 | 60 |
| 35 | 56 | 56 |  |  |  | 60 | 60 |

**Supplementary Table 2:** Heating curve of liquids in common laboratory 1.5 and 2 ml reaction vessels placed in a heat block pre-warmed to 90°C. The increase in temperature was monitored by a thermometer immersed in 500 µl (1.5 ml vessel) and 2000 µl (2 ml vessel) glycerol, respectively. Experiment was performed in triplicates. Mean values were rounded up to the nearest integer.

| **Time (min)** | **Temperature (°C)**  500 µl at 90°C | **Temperature (°C)**  2000 µl at 90°C |
| --- | --- | --- |
| 0.0 | 23 | 23 |
| 0.2 | 26 | 25 |
| 0.3 | 34 | 29 |
| 0.5 | 42 | 34 |
| 0.7 | 49 | 39 |
| 0.8 | 55 | 44 |
| 1.0 | 60 | 48 |
| 1.5 | 71 | 60 |
| 2.0 | 78 | 68 |
| 2.5 | 82 | 74 |
| 3.0 | 84 | 78 |
| 3.5 | 86 | 81 |
| 4.0 | 87 | 83 |
| 4.5 | 88 | 85 |
| 5.0 | 88 | 86 |
| 5.5 | 89 | 87 |
| 6.0 | 89 | 88 |
| 8.0 | 90 | - |
| 10.0 | - | 90 |
